# A fight worth remembering: sharing archival materials in interviews to support recall of ex-mental patient activism

**DOI:** 10.3389/fsoc.2025.1603891

**Published:** 2025-08-13

**Authors:** Danielle Landry

**Affiliations:** ^1^Graduate Program in Sociology, York University, Toronto, ON, Canada; ^2^Canada Excellence Research Chair—Health Equity and Community Wellbeing, Toronto Metropolitan University, Toronto, ON, Canada

**Keywords:** archival research, interviews, qualitative methods, institutional ethnography, mad studies, accessibility, memory, activism

## Abstract

This institutional ethnographic (IE) study of a little-known Ontario-based mad history recounts how, in the 1980s and 1990s, ex-mental patients established a number of social enterprises (also known as consumer/survivor businesses), secured government funding and through these sites, got politically active around issues that impacted their lives. This research poses critical sociological questions about the circulation of activist knowledge-practices and the formation of these businesses as sites of community organizing. Methodologically, IE offers an approach through which I began from the experiences of ex-mental patients while aiming to explore how their activist practices are coordinated trans-locally. By interviewing 42 people who were involved in or supported consumer/survivor businesses and by assembling and digitizing materials from their personal collections, archival collections and the businesses themselves, this work brings into view the central role that consumer/survivor business played in mad people’s activism locally. Formulated at the intersection of mad studies, social movement studies, feminist theories and sociology of knowledge, this study drew on IE interviews using archival data in innovative ways. Pointing to concrete examples, I put forward numerous benefits to using archival materials in interviews to aid participants in recalling events from the not-so-recent past. Arguably, engaging archival materials during interviews can enhance accessibility for populations who are older, experience memory issues, have a history of psychiatric interventions, or for anyone who may benefit from material prompts to resituate them to a particular time and space. Looking through materials alongside participants may serve to initiate discussion and prompt recall, evoking participants’ memories of past events and the meaning they attribute to these, in turn producing richer stories. Doing so may help to ensure key informants are able to make meaningful contributions to sociological research on histories of activism. Talking to participants about archival material can help researchers to make sense of those materials, their connections and sequencing. Additionally, audio and visual materials may bring the contributions of community members who are no longer with us back into dialogue with those who are.

## Introduction

What happens when a collective history being researched rarely appears in formal archives, not having been gathered together in a cohesive, orderly collection? Undoubtedly events occurred and the many people sharing in its collective memory can tell its stories and are connected through shared identity, yet this history is largely absent from archival records. Instead, it appears in the scraps of paper, the newspaper clippings, the photo albums in dusty boxes in the basement of the people who took part in it many years ago. What happens when all the primary sources are scattered between precarious lives, jobs and organizations? What then does it mean to gather these dispersed records—documents, videos, photographs, T-shirts, buttons—as part of the research process, so they may be used to articulate collective memories, knowledges and identities?

Further to this, underscoring presumptions of memory as being a place in our mind where recollections of the past are organized in a coherent, linear fashion, what happens when this collective history is an activist history of groups of mad people? How might interview methods be made accessible when research participants experience recall barriers linked to age and iatrogenic harm^1^ from a lifetime of psychiatric interventions? How might archival materials be used to engage people who share in this history and knowledge, through the interview process?

Millar (2006) emphasizes the importance of archival materials “as evidence, as memory triggers, as touchstones” (125) which are socially mediated and contribute to the development of collective knowledges and identities. Though much more than simply prompts, archival materials can provide important memory cues to elicit recollections, whether those are semantic memories (those that affirm that an event took place), or for individuals who were present at the event depicted in the materials, episodic (remembrance of personally experienced events) or sensory memories. In this way, archives can be tools “used to support the creation, preservation, and resurrection of individual memories and, more importantly, their articulation as part of a shared identity” (2006:126).

This article outlines one institutional ethnographic (IE) study where archival materials—largely gathered from personal and business collections—were introduced and discussed during semi-structured interviews. Six benefits of using a range of archival materials within interviews, to aid participants in recalling events from the not-so-recent past, are put forward. First, introducing archival material in interviews facilitated the gathering together of additional materials. Participants directed me to locate documents corresponding to the stories they told, or they offered to share additional materials from their own personal or business collections. Many of these materials would otherwise never have been shared publicly or preserved digitally. When consent is given to share these materials publicly, the multimedia formats of archival records offer many possibilities for knowledge translation and mobilization, nurturing new forms of public sociology. Second, engaging archival materials during interviews may enhance accessibility for research populations who are older, experience memory issues, have a history of psychiatric interventions, or for anyone who may benefit from material prompts to resituate them to a particular time and space. Doing so aims to ensure key informants are able to make meaningful contributions to sociological research on histories of activism that are not well documented in formal archives. Third, looking through materials alongside participants may serve to initiate discussion and prompt recall, evoking participants’ memories of past events and the meaning they attributed to these. Fourth, it can improve responses to produce richer stories and in the case of institutional ethnographic studies, further develop the mapping of social relations. Fifth, looking through materials from personal collections with participants can help the researcher to make sense of those materials, their connections and sequencing. Sixth, audio and visual materials may also bring the contributions of community members who are no longer with us back into dialogue with those who are.

Object-oriented interview studies have also identified a range of benefits to asking interview participants to engage with “mundane things that make up our everyday lives.” For instance, Owen et al. (2021) indicate that object-oriented interviewing can: provide insight into an individual’s life course, improve autonomy for participants in deciding how the conversation will flow, help participants to untangle complex developments in their lives, aide them to speak openly with emotions and from memory to a greater depth, provide larger quantities of data and lastly, provide cathartic opportunities for the participant to reflect. Harrison et al. (2024) add that objects can become dynamic actors in interviews, materializing the events, accounts and experiences of participants as well as materializing their practices. Objects can also serve as “spatiotemporal ‘anchors’” by situating the interview in relation to other places and times, past, present and future (p. 397). Additionally, Harrison et al. (2024) emphasize that digital objects (i.e., photos, songs) can provide insight into the evolving nature of objects and the meanings people assign to them.

Primarily, this article focuses on interview methods, specifically the practice of engaging archival materials within the interview process, arguing that qualitative interviews outside of the oral history or life story genre could also benefit from bringing archival materials from personal collections and activist ephemera into the interview process. A secondary aim is asserting the early history of consumer/survivor businesses in Ontario as sites of community organizing. By way of an outline, this article began by posing questions of memory and archives, to put forth the idea that archival materials may be beneficial tools to aid in qualitative interviews. Next, the emergence of consumer/survivor initiatives (CSIs) including consumer/survivor businesses in Ontario, Canada will be situated in their historical and political economic context, establishing their link to community organizing, advocacy and activism by current and former mental patients. In the section, ‘Materials and Methods’, the literature on object-based interviewing in the oral history tradition is introduced. I describe how a creative application of Smith’s (2005) institutional ethnography (IE) during the pandemic forms the methodological basis of this research. I account for the materiality of the archival records acquired through this study, consider the relational ethics of engaging these materials with care and reciprocity, and story one example of how materials were gathered through the interview process. In ‘Results’, I provide three concrete examples of how materials were shared or paired within interviews and to what effect. The final ‘Discussion’ section aims to contribute to the literature on sociological research methods by discussing the wider potential application of this method, to better engage with and meet the needs of a range of populations, including but beyond people with mental health histories and older people. I end by reflecting on the impetus to preserve and digitize local material histories of mad activism, particularly in recognition that these material histories and organizations and are at risk of being lost.

### Context

In the 1980s, a few groups of current and former mental patients in Ontario were successful in establishing small businesses akin to social enterprises, or what came to be known as consumer/survivor^2^ businesses. Some of these early businesses included: The Mad Market (established 1980 in Toronto), ABEL Enterprises (established 1983 in Simcoe), A-WAY Express (established 1987 in Toronto) and Fresh Start Cleaning and Maintenance (established 1989 in Toronto)—the last three of which are still in operation in 2025.

Ontario’s political and economic context was shifting dramatically at the beginning of the 1990s. Canada was experiencing a deep recession from 1990 to 1992, followed by high rates of unemployment. The period from the late 1980s to early 1990s marked a significant shift in mental health policy in Ontario (Nelson et al., 2001). To the surprise of many, in 1990 a leftist New Democratic Party (NDP) provincial government took power for the first time in Ontario’s history. This incoming government had a newfound appreciation for consumer participation^3^ in mental health. Survivor-led alternatives to the mainstream mental health system, such as peer support organizations and drop-ins, had been cropping up across the province in the 1980s, mostly operating on shoestring budgets. In addition to advocacy by consumer/survivors, throughout the 1980s, organizations such as the Canadian Mental Health Association (CMHA) National had been working to develop the policy groundwork to support consumer participation (see: Church and Trainor, 1986) and the Graham report in 1988 (Provincial Community Mental Health Committee, 1988) signalled a significant shift to future investment in community-focused mental health services. Then in 1991, Ontario announced $3.1 million in anti-recessionary funding for the Community Mental Health Branch of the Ministry of Health. The branch used the funds to establish the Consumer Survivor Development Initiative (CSDI), now known as Peer Works, citing recent policy recommendations including the Graham Report. CSDI would go on to fund consumer survivor initiatives (CSIs) such as peer-support groups, but also consumer/survivor businesses. From the initial call for proposals, 42 CSIs were selected from 250 applicants to receive provincial funding through the Ministry of Health. The successful CSIs covered all regions of the province, becoming formally recognized as part of the mental health sector in Ontario (O’Hagan et al., 2009).

The Consumer Survivor Development Initiative came about during a time of significant change in the consumer/survivor/ex-patient (c/s/x) movement. More commonly referred to now as the ‘mad movement’, the consumer/survivor/ex-patient (c/s/x) movement emerged during the late 1960s and early 1970s as a loosely organized social movement led by ex-mental patients (Everett, 2000; Morrison, 2005; Reaume, 2021). What began as a decentralized grassroots movement across the Global North, is now international in scope but remains fragmented and loosely coordinated (Logan and Karter, 2022; Morrison, 2005). In the 1990s in Ontario, the c/s/x movement—then in its ‘consumer’ phase—was more reformist and entrepreneurial than earlier movement organizing, adapting to the recession and later, to a conservative provincial government (1995–2003). Adopting new language that reflected an engagement with (rather than strict opposition to) the mental health system, consumers pulled away from more radical organizing, shifting to more service-oriented approaches. Little research has been done on this less radical ‘consumer’ phase of the c/s/x movement or the unique organizational form of the CSI, despite CSIs being hugely innovative, highly effective, and cost-efficient (Shute, 2021).

The study at the center of this article focuses on the advocacy, activism and community organizing which took place within consumer/survivor businesses from 1980 to 2005 in southwestern Ontario. Arguably, despite less radical inclinations during the ‘consumer’ phase of the c/s/x movement, substantial covert and overt forms of advocacy led by consumer/survivors were cultivated within CSIs, including consumer/survivor businesses during this period. Consumer/survivor businesses are small and similar to social enterprises; they operate primarily, though not exclusively in the service sector. A large number of these current and former businesses operate in and around the Greater Toronto Area (GTA), as outlined in Table 1. Though some consumer/survivor initiatives were intentional about documenting their activities, consumer/survivor advocacy has generally not been preserved in conventional archives or databases. Some exceptions to this include the Psychiatric Survivor Archives of Toronto^4^ and a few fonds with the Toronto Metropolitan University Archives.

**Table 1:** Consumer/survivor businesses in Ontario.

	Consumer/Survivor business	Sector	Location
1.	**ABEL Enterprises**	Woodworking	Norfolk county/Simcoe
2.	**A-Way Express**	Courier service	Toronto
3.	Cambridge Active Self Help (C. A. S. H.)	Ceramics	Cambridge
4.	Clerical Express	Secretarial	Kitchener
5.	Crazy Cooks Catering*	Food services	Peterborough
6.	C/S Lawn Care	Landscaping	Etobicoke
7.	Daisy Café	Food services	Windsor
8.	**Fresh Start Cleaning and Maintenance**	Cleaning	Toronto
9.	Garden Delight Juice Bar	Food services	Windsor
10.	The Grill*	Food services	Toronto, on-site at CAMH
11.	Innovative Enterprises	Social enterprise development	St. Catharines
12.	Inspirations Studio (previously Inspirations Women’s Collective)** and Ideas Work Studio	Arts and crafts	Toronto
13.	The Mad Market	Consignment	Toronto
14.	New Look Cleaning	Cleaning	Etobicoke
15.	Our Place	Arts and crafts	Norfolk county/Simcoe
16.	Out of this World Café*	Food services	Toronto, on-site at CAMH
17.	ParcArt	Arts and crafts	Toronto
18.	Parkdale Green-Thumb Enterprises*	Landscaping	Toronto
19.	Prezents of Mind	Arts and crafts	Toronto
20.	Quick Bite Catering and Take-Out	Food services	Brantford
21.	Raging Spoon*	Food services	Toronto
22.	Rainbow Ceramics and Crafts	Arts and Crafts	Hamilton
23.	RecyCLEAN	Cleaning	Oshawa
24.	**Ten Friends Diner**	Food services	Windsor
25.	Wise Choice Café	Food services	Barrie

The 8 businesses indicated in bold remain in operation in 2025. *Working for Change, previously the Ontario Council of Alternative Businesses (OCAB) and prior to that the Consumer Survivor Business Council, is an umbrella organization which operates these businesses solely or in partnership with other organizations, as well as leadership and training programs. See: workingforchange.ca. **Initially established by a CSI as a craft collective for under-housed women, Inspirations Studio now operates as a storefront ceramic studio for women and gender diverse people who experience poverty, marginalization, homelessness or mental health issues.

Though “social enterprises exist on a spectrum” (Ali, 2017), the ‘by and for model’ (Corbière et al., 2019) of consumer/survivor business makes them quite distinct from earlier models of vocational rehabilitation, sheltered workshops and other forms of psychiatric service provider-led programming. This is primarily due to their grassroots origins, egalitarian approach, compensation for workers, and being survivor-led at all levels of the organization. Secure government funding marks consumer/survivor businesses as unique to Ontario and makes it possible to offer a limited number of consumer/survivors much needed accessible job opportunities. This funding structure afforded consumer/survivor businesses the opportunity to fully establish themselves and through these sites, many consumer/survivors found community and got politically active around shared issues that impacted their lives. Arguably, this funding would have the unintended effect of structuring the possibilities for local consumer/survivor/ex-patient movement activities throughout this time period.

There was significant overlap between the aims of c/s/x movement in Ontario and what CSIs including c/s businesses were advocating for because movement leaders often doubled as leaders of these organizations, which in turn became sites where advocacy was incubated. As described by one interview participant, Jennifer Chambers, CSI leaders “were the most visible part of the movement in Ontario” during that time. At their early stages, consumer/survivor businesses were a means to an end for c/s/x organizers. Survivor leaders who took it upon themselves to manage and lead these businesses, such as the late Diana Capponi, were in fact “in the business of changing lives” (Church, 2020). Few survivor business leaders held business, managerial or finance backgrounds which might be more common amongst other forms of social enterprise. In a presentation to the Ontario Association of Social Workers, Dr. Tanya Shute, a past executive director of the Krasman Centre (a CSI peer support drop-in based in Richmond Hill, Ontario), referred to CSIs (of which consumer/survivor businesses are a part) as “born of a movement and accountable to that movement” (Shute, 2021).

There is more to consumer/survivor businesses than their therapeutic potential, or their ability to put a few dollars in community members’ pockets. While these are important aspects, they overlook what motivated establishing consumer/survivor businesses. While CSIs have changed over time, interview participants narrated them as having resistant, even disruptive origins. Having a base of operations during a recessionary period was a valuable resource which survivor leaders could leverage to rally community members and organize around pressing issues facing their community. For example, the Raging Spoon restaurant^5^, a consumer/survivor business with a large space often used for c/s/x community gatherings, once hosted an event where the Parliamentary Assistant to the Ontario Minister of Health, Dan Newman, was invited to hear directly from consumer/survivors about what they felt needed to change in the mental health system.^6^ Hosting the event in a space that was accessible to consumer/survivors, allowed over one hundred community members to attend and participate in the Ministry’s stakeholder consultation.

In the broader literature on social firms or social enterprises for disabled people, generally stated, research questions tend to focus on vocational outcomes, accommodations for workers, employment retention and/or job satisfaction. In the last decade, only a handful of studies on consumer/survivor businesses have been published (Buhariwala et al., 2015; Corbière et al., 2019; Hall and Wilton, 2011; Krupa et al., 2019). None of these recent studies lays out the formation of these businesses as an explicitly activist intervention and site of community organizing. Going back further, over the last three decades there have only been a dozen or so more studies of consumer/survivor businesses (Church, 1996, 1997, 2001; Church and Creal, 1995; Hartl, 1992; Krupa, 1998; Parkes et al., 2002; Shragge and Church, 1998; Trainor et al., 1997; Trainor and Tremblay, 1992). These early studies were more often written up by researchers with some direct connection to consumer/survivor businesses and were more likely to acknowledge advocacy as a trademark of this organizational form. I argue that consumer/survivor businesses in Ontario were sociologically significant sites of community organizing and to miss this point in the recent literature on consumer/survivor businesses is to misunderstand their purpose. Studying the formation of this unique organizational form, particularly in those early years (1980–2005) when advocacy flourished, offers a deeper understanding of how consumer/survivor advocacy was socially organized. In what follows, I introduce this study’s methodology, institutional ethnography, and methods of conducting semi-structured IE interviews, gathering archival materials, and bringing some of these materials into the interview process.

## Materials and methods

Within the fields of oral history and life story or life history research, there are established practices of bringing material objects into the interview process (Plummer, 2001). Object based interviewing, or object-oriented research has been used to study material cultures in family relations and household spaces, often through the use of photographs, personal or domestic items (see, for instance: de Cardoso Sousa, 2021; Green and Luscombe, 2019; Hall, 2019; Wilton, 2008, 2015). Personal possessions have also been incorporated into oral history studies of migration narratives (Cosmini et al., 2018) and in studies of diasporic identities (Ajit, 2015). In object-based interviewing, interviews are sometimes led primarily through a focus on an object itself (Ravn 2022), though some proponents argue for an approach that does not make the object itself the dominant focus (see, for instance: Wilton, 2008). Creative research activities may also be engaged as part of object-based interviewing, such as: freewriting, close looking, collage, line-drawing or mapping (Bates and Fleetwood-Smith, 2025). Reichard’s (2012) study of queer campus activism in 1970s America brought activist ephemera into oral history interviews, which he argues are well-positioned to both aid participants in recalling events and as a producer of content (ephemera revealed through oral history). The meaning of the student-produced ephemera in Reichard’s study was enhanced through oral history, by “capturing the ‘self-understanding’ of those who created it” (2012, p.54) and contributing those self-understandings to the creation of collective memories of queer activism. Reichard’s study highlights the need to preserve and make sense of activist ephemera, particularly amongst marginalized communities whose contributions are not well represented in ‘official’ records.

This article, based on my doctoral research,^7^ is informed by the work of sociologist Dorothy Smith. Smith’s (1978, 1990) and David and Smith (1975) early theorizing was heavily influenced by the women’s movement and to a lesser extent, ex-mental patient organizing. Smith’s ‘*K is Mentally Ill*’ (1978) and *The Conceptual Practices of Power: A feminist sociology of knowledge* (1990) stand out as testament to feminist theorising that challenged core sociological tenets, namely how objectified forms of knowledge get used to oppress women. In *The Conceptual Practices of Power*, Smith draws from her own experiences of psychiatrization (‘Introduction’, 1990) to brazenly challenge the fundamental tenets of psychiatry. Smith’s work stands as feminist sociological cannon with respect to madness, pushing back against women’s psychiatric oppression. Through statistical and text-based analyses, Smith demonstrates to researchers how we might flip the questions that we ask and pay attention to psychiatric practices in order to understand how ruling relations determine women’s experiences as psychiatric patients. Smith’s early publications (1975; 1978) made bold arguments for understanding women’s experiences of the psychiatric system as a political issue.

The methodological framing of this study follows Smith’s institutional ethnography (IE). A historical materialist approach positioned as an alternative sociology “for people” (Smith and Griffith, 2022), or as a method of inquiry, IE starts from research subjects’ every day/night lives and extends beyond that experience as it aims to discover the social. The social can be located in how people’s practices are coordinated trans-locally with the doings of others. Research subjects are not objectified in the process; rather standpoints serve as entry points for discovering and mapping social relations. Embracing a social ontology, IE has two main aims: first, to explore and critique ruling relations in their institutional form to enable people to orient their experience to where they want to go, and second, to build knowledge and methods of discovering ruling relations in contemporary society (Smith, 2005).

IE studies often incorporate textual analysis to examine how relations of ruling operate in the every day/night lives of people in specific institutional settings. IE looks to texts (broadly defined) as a material basis of the discourse that shapes ruling relations. Most institutional ethnographies study present day issues, though some historical institutional ethnographic research exists, including those few that have used a combination of oral history and archival research (Kinsman and Gentile, 2010; Luken and Vaughan, 2005, 2006) or have engaged photographs in interviews (McCoy, 1995).

For this study, which required a creative application of historical institutional ethnography (IE) during the COVID-19 pandemic, I conducted interviews using IE interviewing techniques (Campbell and Gregor 2002; DeVault and McCoy, 2006). The analysis of interview data was also informed by IE. Within this article, I am not presenting a textual analysis of archival materials in the IE sense, or attending to how participants use texts in their work. Instead, I reflect on engaging archival materials during the interview process, to argue for this method’s applicability more broadly, across qualitative interview methods in sociology, beyond oral history and life story research. By working to preserve the archival materials that were shared with me through this study—as I describe in the final discussion—there exists a possibility for later research and analysis of archival materials. Unlike oral history or life story research, recordings of the interviews were not preserved as testimony. IE interviews honor the stories shared by participants, but the study begins rather than ends at this point. Their stories provide valuable insight into how people understand their experiences, motivations, decision making; however, the purpose of IE is to see how people’s work is socially organized—in this case how their activism has been socially organized—so the interview transcript acts as a starting point for analysis, not as an artifact for preservation, nor are they considered the ‘truth’ of the matter. Reflecting on innovative methods in IE is also important for taking IE methodology in new directions, as new factors such as technological innovations and shifting global contexts such as pandemics, require adapting techniques.

In total, I interviewed 42 participants, some on multiple occasions. These were primarily individual interviews, though three small group conversations took place whereby a number of workers or board members from the same workplace were interviewed together at the same time. All were semi-structured interviews using an interview guide as well as, in many cases, the discussion of some form of archival materials. As is the tradition in IE, interview questions focused on the every day/night work people do (or in this case, have done), where work is defined in the broadest sense as any intentional activities that take time and effort. Interviews were conducted over the phone, over Zoom or in person, depending on participant preferences. I spoke with workers from consumer/survivor businesses as well as leaders and board members from consumer/survivor businesses and CSIs during that time period. I also spoke with people who founded or help to found consumer/survivor businesses, civil servants, policy makers, service providers and academics who were involved as allies supporting these organizations in some way. Participants were given the option of remaining confidential or using their real names; the majority chose to use their real names.

The study involved large quantities of archival materials in a wide variety of formats. As part of the interview consent process, I asked people if they had any materials they would like to share as part of the research. I was taken aback by the generosity of community members who were willing to share all sorts of materials^8^ from their personal collections and consumer/survivor business archives. Viewed collectively, these materials tell a story of activism, acts big and small, by consumer/survivors and the important role consumer/survivor businesses played as sites of community organizing.

This study builds on a longer lineage of community histories of mad activism. Gallagher (2021) explains the significance of local grassroots community history projects such as Oor Mad History in Edinburgh and the Survivor History Group in England for documenting the collective actions of service user/survivor movements through the archiving of primary sources. These projects carve out space for survivors to enter into historical research and re-historicize histories of madness (Voronka and LeFrançois, 2022). Projects such as these work to reclaim subjugated histories, challenge authoritative knowledges with counter-discourses developed in community, and create places where survivors can see themselves reflected as agents of change (Gallagher, 2021). Over the years, fruitful exchanges have taken place between Oor Mad History and Mad Studies projects with similar aims in Toronto, Canada, notably the Toronto Psychiatric Survivor Archives and the development of Mad Studies courses rooted in local activist histories (CAPS Independent Advocacy, 2021). Kathryn Church’s extensive work as an independent researcher for consumer/survivor businesses, meticulously documenting their social movement learning, later informed her scholarship in Mad Studies and efforts to secure courses in mad people’s history at Toronto Metropolitan University. Gallagher claims Mad Studies “appears to be predicated on the inquirer identifying as mad, or as a survivor, on the basis of their experience of being a psychiatric patient” (2021, p. 254). While it is true that community history projects often prioritize survivor involvement, and for good reason, Mad Studies does not make this demand. Church herself identifies as an ally to the movement. Inquiries grounded in Mad Studies are, however, rooted in community organizing and refuse apolitical understandings of history. This requires meaningful engagement with mad politics and praxis (Costa and Ross, 2023; Voronka and LeFrançois, 2022).

Central to this project has been an effort to centre and preserve local mad activist history. In thinking about the things that make up this history, my research practice has been guided by a relational ethics of care and reciprocity (Caswell et al., 2021; Ellis, 2016). It has determined how to proceed with, tend to, digitize and (only with permission) share materials. Though used here to generate conversation and evoke memories, I recognize the importance of these objects as invested with significance by the people who shared them. This required careful consideration throughout the data gathering and interview process. Before each interview, I gave thought to who I would be meeting with, which organization(s) they had been affiliated with, and if I had any materials that may be relevant to share with them. An ethics of reciprocity, though arguably impossible to achieve in an inequitable world, aims to create mutually beneficial relationships and research practices (Caswell et al., 2021). This also determined how I proceeded with materials. For anyone who shared physical materials as part of the research, upon return I also shared back those materials in digital format. For some organizations with limited capacity to preserve their own history or utilize materials from the past, having digital copies was particularly beneficial.

### Uncovering materials and making connections

One example, narrated below, describes how archival materials from personal collections were located through the interview process and engaged through ongoing discussions. In some instances, participants directed me to locate documents corresponding to the stories they were telling. In the first of four interviews I conducted with David Reville, he described how businesses that predated the establishment of the Consumer Survivor Development Initiative (CSDI) had secured financial backing.

“*In 1980, The City of Toronto had a little sort of beginning interest in community economic development. And they had a little bit of money to give out. And I think one of the groups they gave money to was On Our Own, which was Don Weitz's group. And he, they had a little business called the Mad Market. And that would have been the first survivor business in Toronto.”* (David Reville, interview participant, 2023).

He suggested I track down evidence of the Mad Market in past issues of Phoenix Rising, a Toronto-based consumer/survivor magazine from 1980 to 1990, as well as reference to it in Irit Shimrat’s book *Call me Crazy: Stories from the Mad Movement* (Shimrat, 1997). He later emailed me, expanding on the City’s early investments in consumer/survivor businesses as aligning with a community economic development (CED) approach:

“*The city had a small CED program and I believe the Mad Market got $ from it. And I believe, too, that A-WAY (87) and Fresh Start (89) also got start-up money from the City and I think that was down to Jacques [Tremblay]. … I don't know how you'd track it down. If I think of it, I will tell you.”* (David Reville, interview participant, 2023).

As he was a past alderman for the City of Toronto prior to the amalgamation of the GTA and a consumer/survivor organizer, I valued David’s insider knowledge. In instances such as these, participants offered suggestions to support the tracking down of relevant historical materials, materials that could fill in or more fully flesh out the stories being told.

In connecting various documents related to the operations of the Mad Market, I was able to trace some of the organization’s advocacy efforts in a weekly newspaper obituary column for Alf Jackson (NOW magazine, August 14–20, Weitz, 1997). In the obituary, Don Weitz writes about how this first survivor business came to be, when he and his friend Alf founded the Mad Market. Too often I found obituaries to be the places where consumer/survivor advocacy was most clearly expressed, in tributes to the lifelong contributions of consumer/survivors who had passed on. Early efforts to form businesses were relatively scrappy. Consumer/survivors crafted make-shift alternatives, largely due to their exclusion from the mainstream labour market. In recalling the early days of the Mad Market, David described in an interview how this business took shape and was involved in local survivor politics and advocacy efforts.

“*Don [Weitz]and his buddy Alf Jackson had a pickup truck. And they, the night before garbage day, they'd go to Rosedale, and they'd pick stuff. And then they, they had a kind of a, they had various arrangements, sometimes they had arrangements with people who ran flea markets, and they'd have a stall. And sometimes they would just set up in a vacant lot. And that went for a number of years. And the Mad Market followed that. And it had one of the people who ran it was a woman named Carol Stubbs that Kathryn [Church] and I knew, and [pause] Carol is in the… [pauses] 1986, I think it was. I hosted a press conference around ECT. And Don and Bonnie Burstow and Carol Stubbs were at that.”* (David Reville, interview participant, 2023).

**Figure 1:**
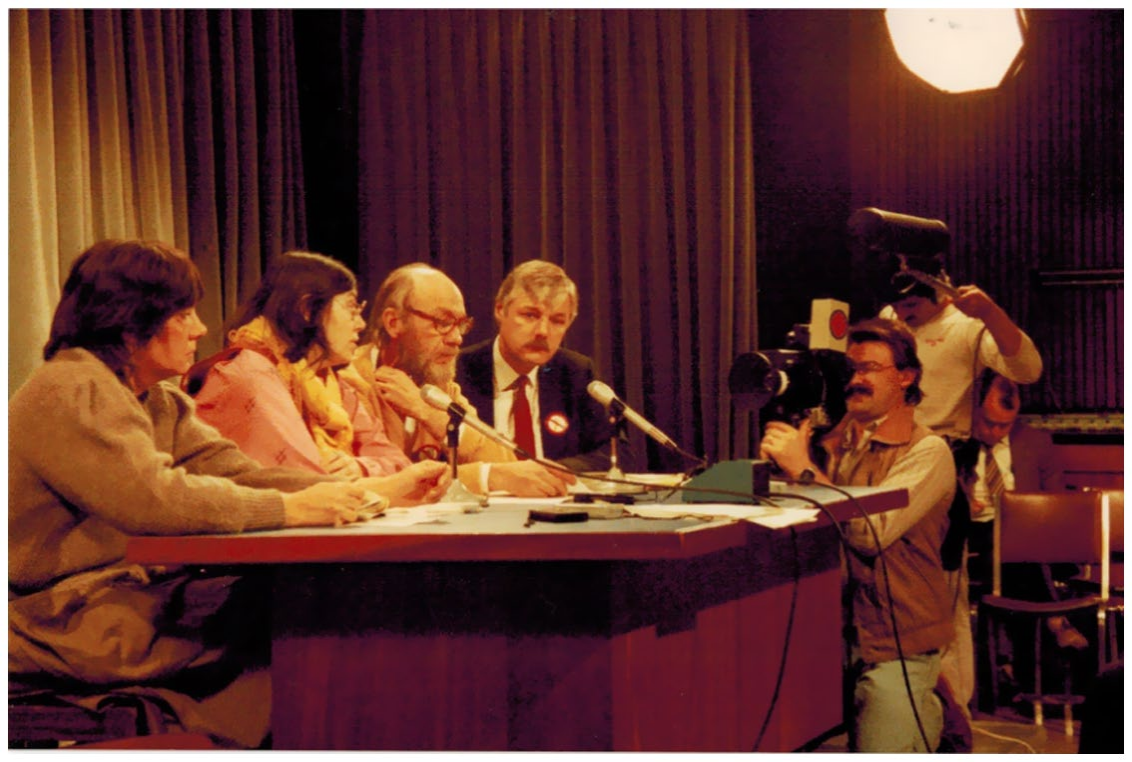
Photograph of a press conference inside the Queen’s Park media room, January 9, 1986. Pictured from left to right: Carol Stubbs (then manager of the Mad Market), Dr. Bonnie Burstow, Don Weitz, David Reville, MPP and two unidentified camera operators. From the personal collection of David Reville, shared with permission.

A few days following this interview, David shared a photograph from his personal collection. The interview had prompted him to look back into this story. The photograph, pictured below, was taken inside the media room in the Legislative Assembly of Ontario, where David then served as a Member of Provincial Parliament. He had organized a press conference on January 9, 1986 for members of the Ontario Coalition to Stop Electroshock, which he then followed up with targeted questions in the House to the Minister of Health. Reviewing Hansard for January 10, 1986, I was able to track David’s exact remarks in the House; he was pressing the Minister to implement the recommendations of the Electro-convulsive Therapy Review Committee and the Gerstein Report (Figure 1).

In recalling this event and by pairing it with Hansard, we were able to see a fuller picture of the press conference, it’s purpose and the significance of the Mad Market’s involvement, as representing the interests of local consumer/survivors. Connecting interviews to historical materials from various sources including personal collections, archival and public records felt like peeling wallpaper. Bit by bit, the underlying image started to appear.

This interview informed me of the intentional sequence of events, as an activist intervention, taken up by multiple stakeholders in the community. It was only through the interview that I knew to track and connect disparate documents, linking them to uncover a fuller story, thanks to participant insider knowledge and involvement in historical events. Even as I located additional documents, such as obituaries, they substantiated the story told in the interview, but also provided more depth, detail and meaning.

Uncovering additional materials was not about triangulating interview data, or prioritizing texts as more reliable sources of data. Instead, this process questions the authority of texts by challenging presumptions that texts are factual and memories are fallible. Arguably, qualitative researchers should never singularly rely on material records or personal memories believing them to be ‘the truth’. Millar (2006) contends, as do I, that archival records are not and should not be regarded in this way, though it might be tempting to point to them as such. Truth, she argues, exists somewhere between the archival records, the memories of participants, of observers and other available evidence. Engaging archival materials with participants through the interview process served to enhance the data gathered by uncovering a fuller picture of past events.

## Results

Arguably, there are many benefits to sharing and discussing archival materials with(in) interviews. Below I describe 3 instances where participants engaged with archival materials in a number of different ways, to the effect of: cultivating access, evoking memories, helping to make sense of materials (by learning about the context, sequencing, and motivations behind actions), and ultimately, producing richer stories about mad activism.

### Cultivating access and prompting recall

In some cases, I sent interview participants archival materials in advance of our meeting. Often this was because I was aware the participant was connected to a specific event, action or organization. Having participants take a quick look at an old photograph was at times enough to bring back a flood of memories, giving them more to talk about and in more detail. In a few cases, I did so to address participant’s concerns that they might not be able to remember much from that time period, as the call for participants indicated the study focused on the period from 1980 to 2005. Asking people to recall events from 30 + years ago can be challenging for anyone, though this may present additional barriers for older people or people who have experienced a lifetime of psychiatric interventions.

When participants indicated concerns, even minor comments such *“I’ll warn you my memory is not what it used to be”* (interview participant), I made an effort to send a few materials, with their consent, over email, to aid in resituating them to that time and place. Sometimes this had a surprising effect. After sending one interview participant an old newspaper article, they began the interview by pointing out how much they had hated the article when it came out for misrepresenting a past leader of the consumer/survivor business where they had worked. This response had me see the article in a new light, as the participant reframed the event of the news coverage and was able to critique the ‘official’ text by reading it in counter-hegemonic ways. In another instance, when I shared newsletters produced by one consumer/survivor business with a past employee of that business, they were able to draw my attention to important parts of documents that I had overlooked.

When I met Graeme Cushing for an interview, I brought along a file folder, as I had already amassed a collection of materials specific to A-WAY Express, a courier company in Toronto, where Graeme has worked for over 30 years. I was aware, from looking at those materials that Graeme had been involved in different forms of advocacy as part of the business. Deferring to the participant’s preference, we chose to wait to look at the file folder together at the close of the interview. During the interview, some of the recollections he had of events were limited: *“I think there was a protest. It was really hot and we got bottled water for people but this is, this is, I really cannot remember.”* (Graeme Cushing, interview transcript, 2023). Even limited information such as this could prove helpful though, as it might mean placing the organization he was representing, A-WAY Express, at an event (in this case, Mad Pride Toronto’s bed push parade) and later locating news coverage or newsletters related to those events.

In the excerpt below from the interview with Graeme, I make reference to a document which he had not yet seen, from a Senate Committee meeting where he was listed as a speaker, in order to prompt his recollection of the range of advocacy activities he had been involved with.

DL: Okay, so, my next question then is: Do you consider any of the work that you have done to be activism or advocacy. Any part of your work or, you know, something associated with your work? How so?

*GC: Well, um I, I think just simply doing your job is a form of advocacy. I think simply day in day out coming as a person with lived experience and doing you're doing a job every single day is testament to that. But also, I mean, there's, there is advocacy. There's, there's, what I'm doing right now is there's public speaking. I've done public speaking before I got up and I've sort of, at AGMs when there's been members of the community there and said, you know, this is why I like to work in A-WAY, this is what it's done for me or that sort of thing. And this is why it's it helps me so much.*

*DL: Apart from AGMs, were there any other events where you were public speaking? So, for instance, I know I noticed in the archives, once you were at a Senate committee meeting for instance, did you speak at that event?*

*GC: I think briefly Yeah. Yeah, I think, it was so long ago, so long ago, but I think I did briefly. When I've spoken before. I spoke about working at A-WAY where there was, there was a gathering down at … have you heard of the Raging Spoon?*

*DL: Yeah, it was such a great space.*

*GC: Yeah, at the Raging Spoon. This was when, this was actually in the Mike Harris days when Janet Ecker she was the minister of Canadian social services. Yes. And we were, I was speaking about my job and she was there and you know, so trying to make an impression on hope-hopefully, her that you know, the importance of work, the importance of maybe allowing people to keep a few more dollars of what they make [overlap] because of ODSP.*

*DL: Exactly, because of the claw back.*

*GC: Yeah, it is terrible. (Graeme Cushing, interview participant, 2023)*

In this example, more specifics became available, such as advocating to ensure their earned income wasn’t clawed back under provisions of the Ontario Disability Support Program (ODSP). The intentions behind this intervention, the targets, and specifics of an entirely different event came up following the prompt. I am very interested in how he as a consumer/survivor identifies being a worker as a form of advocacy in and of itself, since it resists sanist and ableist ideas about what mad people are capable of and challenges normative ideas about valuable workers and benefit recipients. While equally valid, throughout the interview process I had become mindful of a need to push participants beyond the kinds of canned narratives^9^ or stock stories that they rely on from the past, such as the ones they use on behalf of the business. Mentioning the archival material worked to move the conversation beyond a more rehearsed narrative where he has represented himself as a member of the organization and as a person with lived experience.

### Making sense of materials, evoking memories and meaning

In this next example, I describe how archival materials came up for discussion in my interview with Charmaine Frado. Charmaine started up both the Ideas Work Studio and Inspirations Women’s Collective (now Inspirations Studio), which began as a craft collective for un-housed women with mental health histories over 30 years ago. Prior to our interview, I sent Charmaine a few photographs over email. These were group photographs featuring Charmaine and other members of Inspirations which I had located in Working for Change’s archive. Toward the end of our Zoom call, she mentioned the effect of seeing those photographs.

*“We knew years ago that people did not live, live their full life potential with mental health histories, right? Diana [Capponi], that was one of the first things she warned me about. She said, 'Don't expect to know people for very long.' And it was a horrible reality. And so, it's funny when you, when I first got your email, and [another participant] told me all about this, I, I started thinking about people and how many of them are gone. And in fact, your photos, the photos you sent me. I was like,' Oh, they're gone [pointing gestures]. They're gone.' And I thought, 'Oh, my God, this is heartbreaking.' Right?”* (Charmaine Frado, interview participant, 2023)

Though well aware of the shorter life expectancies of consumer/survivors, I had not considered the potential risk in this instance, as Charmaine is only in her mid-fifties. She went on to affirm that this was the reality, and seeing the photographs helped her to remember people. “*You remember your history with the people and you remember them with respect for what they did while they were here.”* (Charmaine Frado, interview participant, 2023).

During the interview, I asked Charmaine about her work to develop a safe house called Edmond Place.

Charmaine spoke about Edmond Wai-Kong Yu, a 35-year-old consumer/survivor whom she had known before his death at the hands of Toronto Police. Following Edmond’s death there was an inquest and his death was ruled a homicide. The coroner’s jury recommendations included the need to develop housing for people in Edmond’s position, specifically people who were homeless, had severe persistent mental health issues and could not access services or were accessing them in ways that were not helpful to them^10^. She described how consumer/survivors in the community gathered together at Parkdale Activity Recreation Centre (PARC) where Edmond had been a regular fixture. People began to meet regularly to plan a safe house project based on the Soteria House model in California. PARC was able to secure some funding to hire someone to coordinate the project, which Charmaine then applied to and was hired to undertake. She referred to this as a “dream project,” though no one knew at the time if it would ever become a reality. She went on to describe the long haul of working on the project for nearly 10 years, overcoming many hurdles securing financing and a building site until they eventually opened Edmond Place in 2010. The redesigned heritage building now offers 29 units of permanent, affordable self-contained supportive housing on a site which had previously been an overcrowded boarding house partially destroyed by a deadly fire.

During the interview she brought out a photo of Edmond that she kept on her desk to this day, despite living in a different city decades later. *“No matter where I go, no matter what I do*—*Edmond goes with me.”* She tearfully stated: *“I firmly believe that there has to be a place in the world for the Edmonds.”* (Charmaine Frado, interview participant, 2023).

Interviews helped to make sense of the material history of consumer/survivor activism, the significance of these materials and people’s connections to them. In the case of Edmond Yu, the public and community outrage following his death led to many forms of activism and a recognition for the need for anti-racism work within consumer/survivor spaces and better cross-movement organizing. Other interview participants also shared documents related to Edmond Yu’s death with me, including a report on Edmond Place, a copy of the Verdict of the Coroner’s Jury, and an anti-racism training manual for workers at one of the consumer/survivor businesses. The training manual includes the case of Edmond Yu’s death, to explain the intersecting nature of racism and sanism. Connecting these various materials through interviews helped to provide context, offering a bigger picture of the sequencing of events and their motivating force.

In this study, I found time and time again how grief, outrage and memory of community members who had died prematurely were drivers for advocacy and collective efforts towards system change. In Charmaine’s case, she dedicated 10 years towards building a safe house in Edmond’s name and continued to carry his memory—and a photographic reminder of it—into her future work. In this way, participants reminded me of the significance of archival materials from personal collections. Discussing materials in interviews served to deepen participant stories and emphasized the meaning and impact of loss in consumer/survivor communities.

### Bringing survivors’ perspectives and knowledge back into the conversation

In a final example, I used recorded footage of Diana Capponi, past ED of the Ontario Council of Alternative Businesses (OCAB) from 2009, speaking as part of a filmed interview for a short web-based documentary. Diana died in 2014. In the footage, Diana narrated how Out of This World Café was created as the first consumer/survivor business (to her knowledge) to be established inside a mental hospital—Canada’s largest mental hospital, the Center for Addiction and Mental Health (CAMH). She describes how it took many years of convincing to get the hospital to divest from their canteen.

The knowledge-practices (Casas-Cortés et al., 2008) of consumer/survivor activists are rarely captured and credited in formal academic or even grey literature. Due to shorter life expectancies (Chang et al., 2011; Hjorthøj et al., 2017), their knowledges are less likely to be documented or preserved before their passing. As part of this study, I interviewed the past CEO of CAMH during that time period, Paul Garfinkel. In my interview with Dr. Garfinkel, I asked him what he recalled about the handover of the canteen business to OCAB (now Working for Change) which subsequently became Out of This World Café. Looking through Working for Change’s archival records, I had seen photos of him at the ribbon cutting ceremony, so I was aware he had been involved, even peripherally, as his Community Relations team managed the handover. His recollection was very limited:

*“I don’t remember it at all. I remember there was a canteen? But I didn't, I don't even associate it with Out of This World Café. I see it as a totally different phenomenon, sorry.”* (Paul Garfinkel, interview participant, 2023)

I also asked him if there were any lasting effects or impacts from the working relationships between CAMH and OCAB. “*Yeah, I would say they gave us an awareness and confidence to do more.*” (Paul Garfinkel, interview transcript, 2023). He went on to describe a number of community involved projects that had been developed at CAMH under his leadership in the years that followed the opening of the café, emphasizing, *“I do not think we would have done that if we had not had the experience with Out of This World.*” (Paul Garfinkel, interview transcript, 2023). He continued to refer to a progression from that positive experience with one consumer/survivor-led business towards less institutional hesitation overall with consumer participation, noting how *“it brings staff along in an important way.”* (Paul Garfinkel, interview participant, 2023).

Towards the end in the interview, with his permission, I played the 2-min video clip where Diana Capponi tells the story of Out of This World Café’s establishment, from her vantage point:

*“I had advocated for a long time that the canteen here should be a business that could help train people and provide employment for folks. The argument back was, that's not really, we're not integrating people back into the community, if they're working here, it wouldn't be healthy for them to work here. And I would always challenge that and say, we need to divide up the economy here. If it's healthy for you, it would be healthy for me. Over time, my understanding is a lot of the volunteers died off. Here at CAMH there was a, an attempt to develop a community development corporation within the institution. And that community development corporation's first initiative was the canteen, and for five years, they paid people cash weekly, under the guise of trainees. … And so, they got into a little bit of trouble with Revenue Canada. And at that point, OCAB was approached and asked if we would assume responsibility or ownership.” (Diana Capponi, filmed interview July 2009, onsite at CAMH)*

Viewing the clip clearly brought back sensory memories for Dr. Garfinkel who acknowledged it was the first time he had ‘seen’ Diana since her passing. He went on to reiterate in a bit more detail:

*“The only thing I knew was about Revenue Canada and the canteen. I was in a role where, you know, where they brought you problems. And that's maybe all you heard about. So I knew they had trouble and then I knew we had this new business. I hadn't linked them actually in my mind. [pause] What she says is completely right. People at one point had hesitation and as I said, by the time of this, hesitation at least for me was gone.”* (Paul Garfinkel, interview participant, 2023).

My decision with this interview to use filmed archival footage was deliberate and specific: to bring Diana’s voice back into the conversation. It’s not that viewing the video changed Dr. Garfinkel’s story, though he was able to expand on what he could remember, elaborating on earlier points. In this care the use of archival material as prompt was not to address access barriers, but it still proved beneficial. Dr. Garfinkel’s response provides insight into higher level decision making and he corroborated the story of a consumer/survivor leader, whose voice would otherwise no longer be part of the conversation.

## Discussion

As previously stated, engaging archival materials in interviews in this way is not about triangulating my data, or seeing any one story or document as ‘the fact’ of the matter. Rather, doing so served to peel away the wallpaper, so to speak, to uncover a fuller picture, to gain a deeper understanding of how consumer/survivor advocacy was socially organized and to center and honour consumer/survivor histories.

Numerous challenges came up throughout this process and are worth noting. Gathering and engaging archival materials from personal collections led to more complex data collection and simply a larger volume of data than originally anticipated. It was time consuming and required gathering additional resources and partners to support the preservation and digitization of archival materials when consent was given. There were also risks that were not fully anticipated, such as in Charmaine’s case when participants, upon seeing old photographs, are struck by how few people remain alive.

Despite these challenges, six benefits to discussing archival materials within qualitative interviews were identified. First, bringing archival materials into view facilitated the gathering of additional materials from participants’ and business’ collections. Many of these materials had been tucked away long ago by people involved in consumer/survivor business and CSI activities. Amidst the closures of many businesses and the precarity of many consumer/survivors’ lives, these multimedia materials may never have otherwise been shared publicly or preserved digitally. When consent is given, the multimedia formats of archival records offer rich possibilities for knowledge translation and mobilization that go well beyond the limits of academic publishing, fostering new forms of public sociology. A second benefit is accessibility, as archival materials can serve as memory aids during the interview. Further, all sorts of people may benefit from resituating themselves in time and place through archival materials prior to speaking on events from the past. Third, some participants were able to speak more concretely or in more detail about their recollections when material histories were shared, aiding to bring memories to the forefront. Fourth, the pairing of archival materials and interviews served to breathe life into the story. Ethnographic narratives become more textured and vibrant when interviews go further in depth and materials can be used to illustrate examples. Fifth, participants can aid the researcher in making sense of materials, by narrating the sequence of events, describing the connections between seemingly disparate documents or by pointing out elements of interest. And lastly, a sixth benefit related to historical research where many of the people involved are no longer alive, is that materials can be used to bring their contributions and perspectives to light.

The central role of an archival institution, according to Millar, “ought to be to seek out the records of its society and make those records *accessible* so that the society may *use* them not just to document events but also to interpret, shape, and articulate memories” (2006:122) [emphasis added]. This paper argues there is a sociological significance to collectively remembering, preserving and sharing mad activist knowledges and histories (Choudhury, 2015), in light of the precarity of mad people’s lives and the political economic systems that erode them. My work aims to take up that imperative in some small way. Arguably, mad and disabled people’s activism alters our understanding of ‘what counts’ as activism, to see every day activism as world making (Dokumaci, 2023). Mad and disabled people must constantly improvise habitable worlds for themselves and institutional ethnographic methodology reminds us there is significance in documenting the complexity of these labours. Sociological studies that center madness and disability are needed in that they serve to expose and disrupt a normative order.

This article contributes to historical research on mad activism by thinking through the possibilities and effects of engaging archival materials in interviews. Narrating three examples above brought into view the kinds of activities local mad activists undertook during the ‘consumer phase’ of the consumer/survivor/ex-patient movement in Ontario. Accounting for consumer/survivor businesses past and present, as this study has done, is important work, in part because this history is at risk of being lost. Table 1 compiled the list of consumer/survivor businesses in Ontario from archival materials and the accounts of interview participants. As indicated, only eight of twenty-five businesses remain. Foregrounding this unique organizational form, I argue these sites played a central role in local consumer/survivor activism throughout the 1990s.

My interview participants were incredibly generous in terms of entrusting me with the material history of these businesses and their community organizing efforts, which has not yet been properly documented in any formal archives. I feel a deep sense of responsibility to honour the community involvement, work and activist contributions of consumer/survivors and those who shared these material histories with me. Through mentorship by mad activists and academics, I have been taught that mad community knowledges and histories belong to those communities (Reville, 2021). So early in the research process, I struck up a partnership with Madness Canada^11^, to secure a permanent home for a digital archive of consumer/survivor business activism^12^. A second consent form was provided to research participants who wanted to see any materials preserved and shared in a publicly accessible format. Being guided by a relational ethics of care means I recognize how precious these materials are and feel a responsibility to see this through, as I co-curate this digital archive with consenting participants. I am grateful that the development of this digital archive will eventually help to bring this little known local mad activist history to light.

## Data Availability

The datasets presented in this article are not readily available because the participants of this study did not give written consent for interview data to be shared, so due to the sensitive nature of the research, supporting data is not available. Requests to access the datasets should be directed to Danielle Landry, dlandry@torontomu.ca.

## Footnotes

^1^By iatrogenic harm, I refer to the range of injuries and harms that can result from psychiatric interventions. These can range from physical and psychological injuries to an erosion of trust in medical professions. Iatrogenic harms may include, but are not limited to, memory loss caused by Electro-Convulsive Therapy (ECT), Post-Traumatic Stress Disorder (PTSD) from pharmaceutical and non-pharmaceutical interventions (i.e., physical or chemical restraint use), and harms that are a by-product of being denied care or receiving poor care from healthcare providers. For more on iatrogenic harm, see: Kaissieh (2023) and Johansson-Everday (2023).

^2^Consumer/survivor refers to self-identifiers of (mental health service) ‘consumer’ and ‘psychiatric survivors’; the blended term indicates all people who either use or refuse mental health services. The language of ‘consumer/survivor’ is used throughout the article as it reflects the self-identifiers embraced at that time and place by research participants and their organizations.

^3^By consumer participation, I am embracing the wide range of similar terms (consumer involvement, patient engagement, etc.). Consumer/survivors had to that point been excluded from representing themselves in mental health policy, research and service provision and were advocating for their right to have input into in these arenas. The term ‘consumer’ emerged out of mental health policy changes in the 1980s and 1990s that redefined ex-mental patients as ‘consumers’ to legitimate their involvement, denoting respect by equating them with customers. Psychiatric survivor, on the other hand, was a self-identifier created by ex-mental patients themselves, drawing attention to harms caused by the mental health system (for more, see: Everett, 2000).

^4^The Psychiatric Survivor Archives of Toronto is not currently accessible to the public. The board is currently rebuilding.

^5^For a number of years, the Raging Spoon operated a bricks and mortar restaurant in an old church at 761 Queen Street West in Toronto. The restaurant served as a physical gathering space in and for the community, where survivors worked and came together around food, as featured in the documentary film *Working like Crazy* (1999). It is now a catering company in the Parkdale neighbourhood of Toronto.

^6^This event was described in a filmed interview with Diana Capponi. It can be viewed in this short web-based documentary [starting at 14:20]: https://www.youtube.com/watch?v=YDy6gROCJ-w (School of Disability Studies, 2009).

^7^The research study *“In the business of changing lives”: Activist knowledge-practices and the founding of consumer/survivor businesses in Ontario* was approved by York University’s Ethics Review Board (Certificate # STU 2021–129).

^8^Items gathered directly from interview participants and consumer/survivor businesses included: films produced by or involving CSIs, radio interviews, c/s business photo albums, loose photographs, newspaper and magazine clippings, books, policy documents, reports, program evaluations, training manuals, political speeches, directories of consumer/survivor initiatives, business plans and proposals, conference proceedings, incorporation documents, fax communications, c/s newsletters, magazines, pamphlets, and other c/s publications, T-shirts, buttons, and more.

^9^By canned narratives or stock stories, I am referring to narratives of the self that people have readily at hand as they have been previously reproduced. These are the kinds of stories that get shored up for a particular audience and may be used strategically, but are limiting.

^10^*“8. The Ministries of Health and Community and Social Services should continue funding for the purchase and construction of new housing for consumer/survivors in Toronto. Such housing should include short-term ‘safe-house’ facilities such as the Gerstein Centre. Rationale: Housing is not always affordable and is difficult to obtain and retain because consumer survivors are not always seen as desirable tenants. Housing is a mental health issue and the absence of decent housing is a major determinant of health.”* Verdict of Coroner’s Jury. 1999, April 16. Coroner of Ontario.

^11^Madness Canada: https://madnesscanada.com/.

^12^The digital archive is currently in development with a provisional launch date of 2026.
